# Height Fluctuations and Surface Gradients in Topographic Measurements

**DOI:** 10.3390/ma16155408

**Published:** 2023-08-01

**Authors:** Julie Lemesle, Clement Moreau, Raphael Deltombe, Joseph Martin, François Blateyron, Maxence Bigerelle, Christopher A. Brown

**Affiliations:** 1 U.R Concept, 59300 Valenciennes, France; 2 Valutec, Univ. Polytechnique Hauts-de-France, CEDEX 9, 59314 Valenciennes, France; 3 Univ. Polytechnique Hauts-de-France, CNRS, UMR 8201-LAMIH-Laboratoire d’Automatique de Mécanique et d’Informatique Industrielles et Humaines, F-59313 Valenciennes, France; 4 Digital Surf, 25000 Besançon, France; 5 ESSILOR, Centre Innovation et Technologies CIT3, 94000 Creteil, France; 6 Surface Metrology Laboratory, Worcester Polytechnic Institute, Worcester, MA 01609, USA

**Keywords:** fluctuation, gradient, roughness, topography

## Abstract

Topographic maps are composed of pixels associated with coordinates (*x*, *y*, *z*) on a surface. Each pixel location (*x*, *y*) is linked with fluctuations in a measured height sample (*z*). Fluctuations here are uncertainties in heights estimated from multiple topographic measurements at the same position. Height samples (*z*) are measured at individual locations (*x*, *y*) in topographic measurements and compared with gradients on topographies. Here, gradients are slopes on a surface calculated at the scale of the sampling interval from inclination angles of vectors that are normal to triangular facets formed by adjacent height samples (*z* = *z*(*x*, *y*)). Similarities between maps of gradients logs and height fluctuations are apparent. This shows that the fluctuations are exponentially dependent on local surface gradients. The highest fluctuations correspond to tool/material interactions for turned surfaces and to regions of maximum plastic deformation for sandblasted surfaces. Finally, for abraded, heterogeneous, multilayer surfaces, fluctuations are dependent on both abrasion and light/sub-layer interactions. It appears that the natures of irregular surface topographies govern fluctuation regimes, and that regions which are indicative of surface functionality, or integrity, can have the highest fluctuations.

## 1. Introduction

Surface topographies are important for understanding physical, chemical, and biological phenomena and surface creation in all manufacturing processes. Krolczyk et al. [1] show the importance and the influence of manufacturing process parameters on surface functionalities through surface topographies. Value creation in manufacturing industries derives from providing desired functionality and aesthetics for customers and stakeholders. These functionalities and aesthetics can be related to surface topographies. Evaluations of surface topographies, which are necessary for evidence-based product and process design, quality control in manufacturing, and for advancing science, start with topographic measurements. These generally consist of hundreds of thousands to millions of individual height samplings located in regular, spatial arrays. Evaluating the quality of these measurements is key to evaluating topographies and understanding how they influence functionality and are influenced by processing.

Recently, Lemesle et al. [2] showed in a top-down study of fluctuations how location specific fluctuations, interpreted as uncertainties, in height measurements can be estimated from multiple measurements at the same position. Pixel height is observed on the same surfaces as the current study, measured by White Light Interferometer (WLI) and Scanning White Light Interferometer (SWLI). The height evolutions, called fluctuations in this paper, came from a multitude of measurement-specific conditions and sources, such as measurement apparatus settings, surface type, measurement environment or operator sensitivity, as shown in Figure 1 of Lemesle et al. [2]. More precisely, as interferometry involves speckle patterns, the term ‘fluctuation’ is used to express the consequences of the stochastic variations in the domain of speckle patterns produced by partially coherent light. As defined by G. Parry [3], measurements of rough surfaces by interferometry introduce phase fluctuations consisting of large numbers of scattering regions and lead to complex amplitude fluctuations which are normally distributed. The magnitude of the phase fluctuations determines the mean of these variations. Optically rough surfaces give zero mean complex amplitude fluctuations.

Topographic characterizations, like roughness, R_a_ or S_a_, can be important indicators of the value of manufactured products. Technically, roughness parameters refer to a class of topographic characterization parameters which are calculated from bandwidth-limited data. Roughness parameters are only technically meaningful when the limits of bandwidth-defining roughness are stated.

Many phenomena can be related to topographic interactions. Value in surface metrology research, as well as measurement and analysis of topographies, can be derived from discoveries of strong functional correlations [4] with processing or performance, as well as from confident discrimination of surfaces that were produced or that perform differently [5]. These value-adding abilities depend on adequate measurements at pertinent scales and appropriate statistical treatments of characterizations that are geometrically appropriate for the topographically related phenomena of interest [5].

Concepts studied here are designed to help understand topographic contributions to location specific fluctuations. Industrial processes, such as turning, sand blasting, and grinding, can elucidate these concepts.

The manufacturing process can include a range of parameters, such as temperature, pressure, and velocity. Królczyk et al. [6] highlighted this and explained that process conditions impact topographic formation, and that topographies can be described by combinations of the most relevant topographic characterization parameters. In short, each parameter could be used to describe a surface feature type related to surface functionality. Some papers study parallels between manufacturing processes and roughness parameters from ISO 25178 [7]. Other papers compare manufacturing process variables directly with surface performance without understanding the topographies.

In addition, multiscale characterizations, which are not limited to any selected bandwidths, multiscale analyses like regression and univariate ANOVA [8], and bootstrap methods could offer insight. These studies need topographical characterizations based on a good understanding of the fundamental interactions in the process, adequate resolutions, and sufficient data for stable statistics.

Surfaces can be measured by a wide range of optical instrument types, such as interferometers, focus variation, and confocal microscopes. These instruments use different technologies to sample heights of surfaces at intervals. Each of these technologies achieves its particular resolutions though its surface sensing technologies which are based on different physical principles. Developing specific, bottoms-up methods from physical principles that can determine uncertainties for each type of device is problematic because each combination of measurement system and measurand can have their own idiosyncrasies. A generic, top-down method using fluctuation-based uncertainties in height measurements at each location, with its local geometric properties, is a better way to estimate uncertainties.

Saraç et al. [9] used a ‘top-down’ strategy and experimentally investigated the potential sources of errors in WLI. They distinguished ‘real time methods’ and ‘post-processing methods’ to treat the correlogram and test the uncertainties by a standard deviation of 25-repeated map by using time series analysis. ‘Real time methods’ deliver the depth map of the surface immediately after finishing the scan, whereas ‘post-processing methods’ need the whole dataset for the analysis and begin the evaluation immediately after the scan. Another approach to surface roughness uncertainties is made by Mills [10], where the roughness of road pavements in Kansas was simulated using hierarchical Markov Chain Monte Carlo methods. It was then possible to reflect prevailing roughness conditions without neglecting uncertainty.

Even with precautions and careful analyses, one wonders if it is possible to guarantee results of studies on the influence of measurement data sets on dispersion. The weakness of this vision is the determination of the reliability of the data set. More precisely, the quality of topographic measurements needs to be quantified. Even in cases of excellent discrimination capacities of certain analyses, it is unclear whether differences should be attributed to the topographies or to measurement fluctuations. This is why it makes sense to assess fluctuation-based uncertainties in measurements in order to evaluate their influence on discrimination or correlation results.

A general lack of terminology in the literature is observed regarding the characterization of surface features, while ISO 8785 [11] shows a simplified view of a large surface feature range. A similar observation is possible for gradients and a multiple terminologies and descriptions that can be used to describe surfaces. Gradients are scalar representations of spatial variations of heights (*z*) between one location and its neighbors. Gradients, as mentioned above and demonstrated below, are calculated from normal vectors obtained from triangular facets fitted to adjacent height samples. Gradients, as used here, are sometimes referred to as slopes on a surface. Gradients are calculated here at each location (*x*, *y*) on the surface topography and are used to generate gradient maps.

The objective of this work which to determine the extent to which gradients could be origins of fluctuation-based uncertainties in optical measurement of topographies. This extends a previous top-down study [2]. Fluctuation-based uncertainties are indicated by fluctuations in height measurements (*z*) repeated at a single location (*x*, *y*). Gradients are a local geometric property of a measurand. This paper examines relations between height fluctuations and local gradients at any location on topographic measurement maps.

This is a new approach to fluctuation quantification of topographic measurements. It can increase the value of surface metrology through insights into measurement repeatability, and for finding useful functional correlations and discrimination, which also depend on using pertinent scales and topographic characterization parameters [3]. Lack of repeatability, as indicated by large fluctuations, can impact these abilities.

In optical surface metrology, the optical properties of measurands, which include height gradients, can influence measurement quality. The measurand and physical phenomena of the measurement apparatus are linked in terms of influencing measurement quality due to the point focus detection criteria compared to the measurement technologies used. Height fluctuations and height gradients acquisition therefore depend on the physics used in the measurement systems. At a certain scale, resolution depends on the type of optical properties of surface features, such as brightness and transparency. All measurement systems are not able to measure the same information. At the same scale and resolution, it is better to select an apparatus according to its ability to measure this type of surface with low height fluctuation. Similar reasoning could be applied when strong surface gradient must be measured.

In addition to the scale, surface resolution has an impact on the calculation of some parameters. In a nutshell, the resolution of each measurement apparatus depends on the measuring scale, which in turn depends on the surface being measured. For instance, slope as characterized by the *S_dq_* parameter is based on triangular facets carried by the surface topography point heights with some smoothing. *S_dq_* results depend on sampling intervals, although ISO 25178 does not yet require these to be reported with *S_dq_* [12].

However, one of the most important criteria is the manufactured surface topography. Due to geometric and optical properties at each location, an apparatus’ response to height could depend on the numerical aperture, lateral resolution, or light reflection. For example, the surface reflectivity plays a crucial role in shaping the white light correlogram by determining which wavelengths of light are reflected or absorbed by a surface. High reflectivity leads to a correlogram with strong correlations across the spectrum, while low reflectivity results in reduced correlations for certain wavelengths. More precisely, reflectivity can influence white light interferograms by affecting the intensity, contrast, and visibility, and potentially introducing phase shifts in the interference fringes. Gross et al. [13] demonstrated that the statistical error of each measurement location depends on the brightness of the associated speckle; a dark speckle gives a more uncertain measurement than a bright one. If the brightness is lower than the camera’s noise threshold, the measurement fails completely, and an outlier appears that leads to a NaN value. Pavel Pavliček and Ondřej Hýblt [14] showed that, under both theoretical considerations and experimental ones, the measurement uncertainty caused by the surface roughness depends on the intensity of the individual speckle: the brighter the corresponding speckle, the more precise the measurement. They also showed that distribution of the measurement error caused by surface roughness depends on the roughness itself.

Local material properties can also affect the amplitude of fluctuations and are principally governed by fluctuations in the refractive index [15]. More precisely, refractive index fluctuations refer to the variations in the refractive index of a material or medium over space or time. In an ideal situation, the refractive index of a material is considered constant and uniform. However, in reality, many factors can cause the refractive index to fluctuate. These fluctuations can occur due to variations in temperature, density, composition, or any other physical parameter that influences the optical properties of the medium and affects the shape of the white-light correlogram due to the first-order chromatic dispersion of the refractive index [16].

Landscapes could be described by many maps, including heights, gradients, and grey levels. These maps can be evaluated by correlating them and through localized measurement uncertainty maps. The aim here is not to quantify the uncertainties of single surfaces directly but rather to link the gradients and height fluctuations from a series of surface measurements for an uncertainty deduction.

The methodology proposed in this paper here is summarized:

1. Measuring n times the same surface zone;
2. Calculating the height standard deviation (fluctuations) and the mean gradient at each pixel from the set of n maps;
3. Generating fluctuation and gradient maps;
4. Plotting fluctuations/gradients graphs;
5. Finding a correlation between fluctuations and gradients by using a new developed tool, named Bounded Bivariate Density (B^2^D) plotting method (2D or 3D view).

## 2. Materials and Methods

### 2.1. Choice of Surfaces

The method is applied on the same surfaces analyzed in [2]: one turned surface, two sandblasted surfaces and two zones of a biplane, multilayered ophthalmic lens (Essilor, Creteil, France).

**Figure 1:**
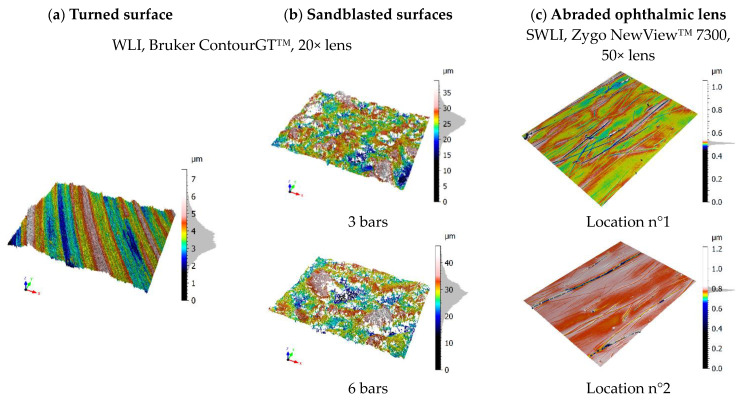
Measured topographies: turned (**a**), sandblasted under a pressure of 3 and 6 bars (**b**), and two zones of the abraded ophthalmic lens (**c**) [2].

These surfaces were selected as representative of surface topographies for application of the following method. The turned surface (Figure 1a) was machined at 120 m/min with a tungsten carbide D-type insert on an aluminum alloy AU4G part. It has periodic motifs and multiscale topographies oriented in the cutting direction. The sandblasted surfaces (Figure 1b) were created by propelling corundum particles at 3 and 6 bars on aluminum alloy AU4G parts for 60 s. These surfaces have isotropic, complex multiscale topographies. Two surfaces were studied to observe the influence in changing the process pressure on the surfaces. Finally, the third surfaces (Figure 1c) were obtained by abrading an ophthalmic lens with an abrasive media for 300 cycles (150 cycles per minute). In a nutshell, this ophthalmic lens is studied because it includes distinct deep scratches, nano scratches on the surface bulk, and pits.

To sum up, a wide choice of surface topographies might be analyzed but only three types of surfaces were selected to perform the analysis because of their local characteristics, such as the gradient, or their particular functionalities, such as transparency. These three types of surfaces are used to:

- Quantify the role of optical properties on the nature of fluctuations (transparent and opaque ophthalmic lens).
- Quantify the influence of directional gradients on fluctuations (isotropic for sandblasting surfaces and anisotropic for tooled surfaces).
- Check the repeatability of fluctuation estimation by taking two different locations of the same ophthalmic lens.
- Quantify the role of roughness amplitude on fluctuation amplitude (two sandblasting surfaces with S_a_ of 2.8 µm for a sandblasting pressure of 3 bars and S_a_ of 3.9 µm for a sandblasting pressure of 6 bars).

### 2.2. Measurement Methods

The turned and sandblasted surfaces were measured on a WLI (Bruker Contour GT™) with a 20× magnification, a numerical aperture of 0.46, an optical lateral resolution of 0.7 µm, and a sampling interval of 0.49 µm. The abraded ophthalmic lenses were measured on a SWLI (Zygo NewView™ 7300) with 50× magnification, a numerical aperture of 0.55, an optical lateral resolution of 0.52 µm, and a sampling interval of 0.22 µm.

A study on roughness parameters (S_a_, S_al_, S_tr_, S_dr_, S_pd,_ and S_pc_) is presented in Appendix B. Each parameter was calculated on each surface presented here.

### 2.3. Bounded Bivariate Density (B*^2^*D) Plotting Method

#### 2.3.1. Calculation of Fluctuations

The method for fluctuations calculation proposed by Lemesle et al. [2] is summarized here. Measured surface topographies consist of amplitudes, *z_i,j,n_* where (*i*, *j*) are the coordinates of a surface location and *n* corresponds to the *n*th map of size (*I*, *J*) from the set of maps *M*. Three kinds of representations of fluctuations are calculated:

1. Mean map, *μ_i,j_* (Equation (1)):

(1) $$\mu_{i,j}=\frac{\sum_{n=1}^{n_{i,j}}z_{i,j,n}\delta_{i,j,n}}{\sum_{n=1}^{n_{i,j}}\delta_{i,j,n}}\;\;\mathrm{with}\;\;\delta_{i,j,n}=\left\{\begin{array}{c} 1\;\;\;\mathrm{if}\;\;z_{i,j,n\;}\neq \phi \\ 0\;\mathrm{else} \end{array}\right.$$

2. Standard deviation map, *σ_i,j_*, calculated from the *n* measured maps (Equation (2)):

(2) $$\sigma_{i,j}=\sqrt{\frac{\sum_{n=1}^{n_{i,j}}{\left(z_{i,j,n}-\mu_{i,j}\right)}^{2}\delta_{i,j,n}}{\sum_{n=1}^{n_{i,j}}\delta_{i,j,n}}}\;\;\mathrm{and}\;\;\sigma_{i,j}=\phi \;\;\mathrm{if}\;\;n_{i,j}\;<\;2$$

3. Normalization of the standard deviation, $\hat{\sigma_{i,j}}$, (Equation (3)), by dividing by the root mean square roughness parameter for the entire measured surface, $S_{q}$, which is the second statistical moment, standard deviation, or variance of the surface heights (Equation (4)):

(3) $$\hat{\sigma_{i,j}}=\frac{\sigma_{i,j}}{S_{q}}$$

(4) $$S_{q}=\frac{1}{N}\sum_{n=1}^{N}S_{q,n}\;\;\mathrm{with}\;\;S_{q,n}\sqrt[{}]{\frac{\sum_{i=1}^{I}\sum_{j=1}^{J}{\left(z_{i,j,n}-\Pi_{d,i,j,n}\right)}^{2}\delta_{i,j,n}}{\sum_{i=1}^{I}\sum_{j=1}^{J}\delta_{i,j,n}}}$$

where *Π_d,i,j,n_* is the polynomial of degree d which best fits to the surface by least squared interpolation on heights *z_i,j,n_* and removes the form from the surface.

#### 2.3.2. Gradient Maps

The idea here is to study the relations of a height, aka pixel, at a location (*i*, *j*) with its close neighbors. Spatial scales must be decorrelated as much as possible. Only the adjacent points are used to compute the gradient.

The computation of the surface gradient in topography is standardized by the ISO 25178:2 standard [7] in which the calculation of the Root Mean Square Gradient, $S_{dq}$, is proposed (Equation (5)).

(5) $$S_{dq}=\sqrt{\frac{1}{A}\iint_{A}^{}\left[{\left(\frac{\partial z\left(x,y\right)}{\partial x}\right)}^{2}+{\left(\frac{\partial z\left(x,y\right)}{\partial y}\right)}^{2}\right]dxdy}$$

Blateyron [17] proposed to associate two plots which represent the *z* normal distribution on each triangular facet of the surface mesh and the orientation of the facet in the (*x*, *y*) plane. He thus showed the great richness of looking at the probability density of the local gradients. The computation is described in the Surfstand project proposed by Blunt et al. [18], who used a seven-points Lagrange interpolation. This proposition was to be extended to 3D by Dong [19] based on an initial idea by Chetwynd [20] (Equation (6)).

(6) $$S_{dq}=\sqrt{\frac{1}{\left(M-6\right)\left(N-6\right)}\sum_{j=4}^{N-3}\sum_{i=4}^{M-3}\rho_{i,j}^{2}}$$

(7) $$\rho_{i,j}={\left\{\begin{array}{c} {\left(\frac{1}{60∆x}\left[-\eta \left(x_{i-3},y_{j}\right)+9\eta \left(x_{i-2},y_{j}\right)-45\eta \left(x_{i-1},y_{j}\right)+45\eta \left(x_{i+1},y_{j}\right)-9\eta \left(x_{i+2},y_{j}\right)+\eta \left(x_{i+3},y_{j}\right)\right]\right)}^{2}\\ +{\left(\frac{1}{60∆y}\left[-\eta \left(x_{i},y_{j-3}\right)+9\eta \left(x_{i},y_{j-2}\right)-45\eta \left(x_{i},y_{j-1}\right)+45\eta \left(x_{i},y_{j+1}\right)-9\eta \left(x_{i},y_{j+2}\right)+\eta \left(x_{i},y_{j+3}\right)\right]\right)}^{2} \end{array}\right\}}^{\frac{1}{2}}$$

However, the use of this interpolation will cause some problems. This intercorrelation strongly correlates the errors on nine points (3 × 3 grid). However, the fluctuations, in particular with high local surface gradients where the points are calculable, i.e., without NaN, especially for the optical methods, are researched. The probability of having one non-measured point in nine, or in twelve, allowing for an interpolation, becomes high and makes the interpolated gradient not calculable. This is highlighted by Gomez et al. [21]: by using a denoising technique, i.e., a 3 × 3 pixel denoising filter, the nine neighboring pixels will be correlated, and the number of uncorrelated image points P will be reduced by a factor of nine. Using the ISO 25178 method, this will lead to correlating 49 points in this study (Equation (7)). Therefore, the idea is not to obtain a smooth gradient which will be well able to characterize a surface [22], but a gradient which can capture the noise. As the noise has fractal characteristics, a method which characterizes it must be retained. Ungar et al. [23] showed that triangulation captures the fractal aspect with optical methods. As a consequence, the computed gradient will be the gradient of the elementary facet. Lemesle et al. showed that computing the gradient on a facet allows for a better description of the physics of failure at very small scales [24].

**Figure 2:**
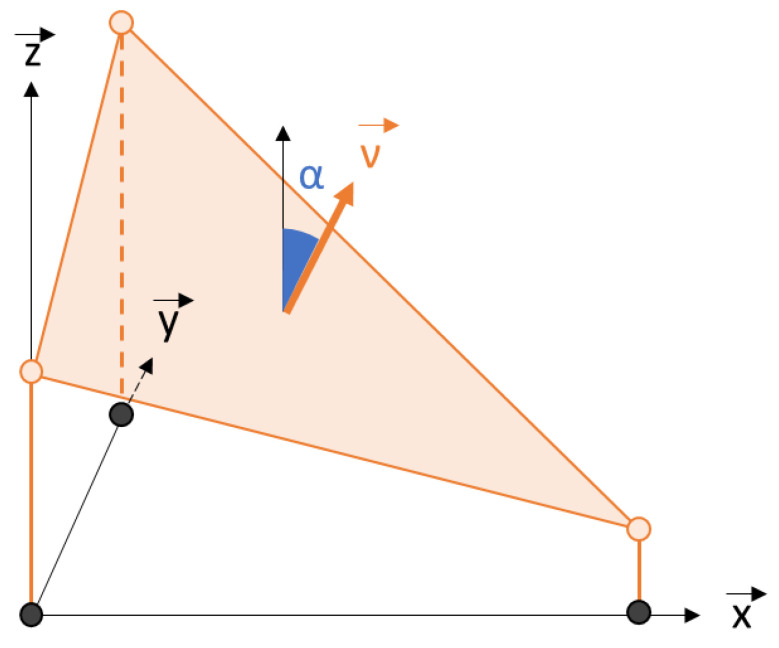
Spatial representation of gradient given by 3D triangular facet obtained from surface mesh inspired by Blateyron [17].

The *z* gradient, i.e., the gradient of the normal (*ν_i,j,n_*) to the surface of the plane (Figure 2), is noted *∇_i,j,n_* (Equation (8)). This gradient is here equal to the *z* normal because it is assumed that the fluctuations will depend on the gradient. The maximum normalized gradient is therefore limited to 1, which corresponds to an infinite gradient. The mean gradient, $\hat{\nabla_{i,j}}$, is obtained by averaging the *n* elementary maps, including NaN, which is described by Equation (9).

(8) $$\nabla_{i,j,n}=\tan\alpha_{i,j,n}=\tan\left(\arccos\left(\vec{z}\cdot \vec{\nu_{i,j,n}}\right)\right)\;\mathrm{with}\;\left\|\vec{z}\right\|=1\;\;\;\;\mathrm{and}\;\;\;\;\left\|\vec{\nu_{i,j,n}}\right\|=1$$

(9) $$\hat{\nabla_{i,j}}=\sum_{n=1}^{n_{i,j}}\frac{{\left(z_{i,j,n}-\mu_{i,j}\right)}^{2}}{n_{i,j}}\;\;\mathrm{with}\;\;z_{i,j,n}\;\neq \;\phi \;\;\mathrm{and}\;\;n_{i,j}=\sum_{n=1}^{n_{i,j}}1;\sigma_{i,j}=\phi \;\;\mathrm{if}\;\;n_{i,j}\leq \;2$$

The moments *Q_q_* of all gradients based on the empirical probability density of *∇_i,j,n_* can be calculated. This probability density is noted (Equations (10) and (11)). The moments $\hat{Q_{q}}$ of the mean gradient map based on the empirical probability density of $\hat{\nabla_{i,j}}$ can be calculated. This probability density is noted as $p_{\hat{\nabla }}$ (Equations (12) and (13)).

(10) $$P\left(\nabla_{1}\leq \nabla \leq \nabla_{2}\right)=\int_{\nabla_{1}}^{\nabla_{2}}p_{\nabla }\left(x\right)dx$$

(11) $$P\left(\nabla \leq Q_{q}\right)=\int_{-\infty }^{Q_{q}}p_{\nabla }\left(x\right)dx=q$$

(12) $$P\left(\hat{\nabla_{1}}\leq \hat{\nabla }\leq \hat{\nabla_{2}}\right)=\int_{\hat{\nabla_{1}}}^{\hat{\nabla_{2}}}p_{\hat{\nabla }}\left(x\right)dx$$

(13) $$P\left(\hat{\nabla }\leq \hat{Q_{q}}\right)=\int_{-\infty }^{\hat{Q_{q}}}p_{\hat{\nabla }}\left(x\right)dx=q$$

A major interest is calculating fluctuations statistics by gradient class. The entire range of values is divided into a series of equal intervals in log scale. However, from preliminary studies, it is possible to build a homogeneous repartition of the fluctuations if the interval is 0.01 by performing the variable change described by Equation (14). A set of *k* − 1 continuous intervals is therefore obtained.

(14) $$\left[{log}_{10}\nabla_{1},{log}_{10}\nabla_{2}\right],\left[{log}_{10}\nabla_{2},{log}_{10}\nabla_{3}\right],\ldots ,\left[{log}_{10}\nabla_{k-1},{log}_{10}\nabla_{k}\right]\;\mathrm{with}\;\;{log}_{10}\nabla_{k}-{log}_{10}\nabla_{k-1}=0.01$$

#### 2.3.3. B^2^D Plot

Bounded Bivariate Density (B^2^D) involves plotting the bivariate density of both gradient and individual standardized fluctuations. The individual standardized fluctuations, *Se_i,j,n_*, is calculated at each location (*i*, *j*) of an elementary measured map and is described by Equation (15). B^2^D is constructed with the pairs (*Se_i,j,n_*, *∇_i,j,n_*) and the 2D probability density function, $p_{\nabla ,Se}$, defined by Equation (16).

(15) $${Se}_{i,j,n}=\frac{z_{i,j,n}-\mu_{i,j}}{Sq}$$

(16) $$P\left(\nabla_{1}\leq \nabla \leq \nabla_{2},{Se}_{1}\leq Se\leq {Se}_{2}\right)=\int_{\nabla_{1}}^{\nabla_{2}}\int_{{Se}_{1}}^{{Se}_{2}}p_{\nabla ,Se}\left(x,y\right)dxdy$$

#### 2.3.4. Fluctuations/Gradient Graphs

From these computations defined previously, several graphs can be constructed.

- Global standardized fluctuations versus Gradients

The graphs of statistics based on for each gradient interval defined by Equation (14) are plotted. The density probability function, $\hat{\sigma_{i,j}}$, is defined according to Equations (17) and (18).

(17) $$P\left(\hat{\sigma_{1}}\leq \hat{\sigma }\leq \hat{\sigma_{2}}\right)=\int_{\hat{\sigma_{1}}}^{\hat{\sigma_{2}}}p_{\hat{\sigma }}\left(x\right)dx$$

(18) $$P\left(\hat{\sigma }\leq \hat{Q_{q}}\right)=\int_{-\infty }^{\hat{Q_{q}}}p_{\hat{\sigma }}\left(x\right)dx=q$$

Several conditions on the density function can be built by class of gradient, indexed by *k* (Equation (19)) and their associated moments {$\mu_{\hat{\sigma }}$, ${Q50}_{\hat{\sigma }}$, ${min}_{\hat{\sigma }}$, ${max}_{\hat{\sigma }}$, ${Q5}_{\hat{\sigma }}$ and ${Q95}_{\hat{\sigma }}$}.

(19) $$P\left(\hat{\sigma_{1}}\leq \hat{\sigma }\leq \hat{\sigma_{2}},\;\left[{log}_{10}\nabla_{k},{log}_{10}\nabla_{k+1}\right]\right)=\int_{{\hat{\sigma }}_{1}}^{{\hat{\sigma }}_{2}}p_{\hat{\sigma },k}\left(x\right)dx$$

The six following graphs can therefore be plotted:

(20) $$\left({log}_{10}\nabla_{k},\left\{\mu_{\hat{\sigma },k},{Q50}_{\hat{\sigma },k},{min}_{\hat{\sigma },k},{max}_{\hat{\sigma },k},{Q5}_{\hat{\sigma },k},{Q95}_{\hat{\sigma },k}\right\}\right)$$

- Individual standardized fluctuations versus Gradients

This graph seems to be similar to the graph described by Equation (19) but it is a complementary information. Instead of taking the *I × J* values of $\hat{\sigma_{i,j}}$, i.e., on the entire set of measurements, to estimate the probability density function, the *I × J × n* values of standardized fluctuations, *Se*, will be taken (Equations (21) and (22)).

(21) $$P\left({Se}_{1}\leq Se\leq {Se}_{2},\;\left[{log}_{10}\nabla_{k},{log}_{10}\nabla_{k+1}\right]\right)=\int_{{Se}_{1}}^{{Se}_{2}}p_{Se,k}\left(x\right)dx$$

(22) $$\left({log}_{10}\nabla_{k},\left\{{log}_{10}\left|\mu_{\hat{\sigma },k}\right|,{log}_{10}\left|{Q50}_{\hat{\sigma },k}\right|,{log}_{10}\left|{min}_{\hat{\sigma },k}\right|,{log}_{10}\left|{max}_{\hat{\sigma },k}\right|,{log}_{10}\left|{Q5}_{\hat{\sigma },k}\right|,{log}_{10}\left|{Q95}_{\hat{\sigma },k}\right|\right\}\right)$$

## 3. Results

**Figure 3:**
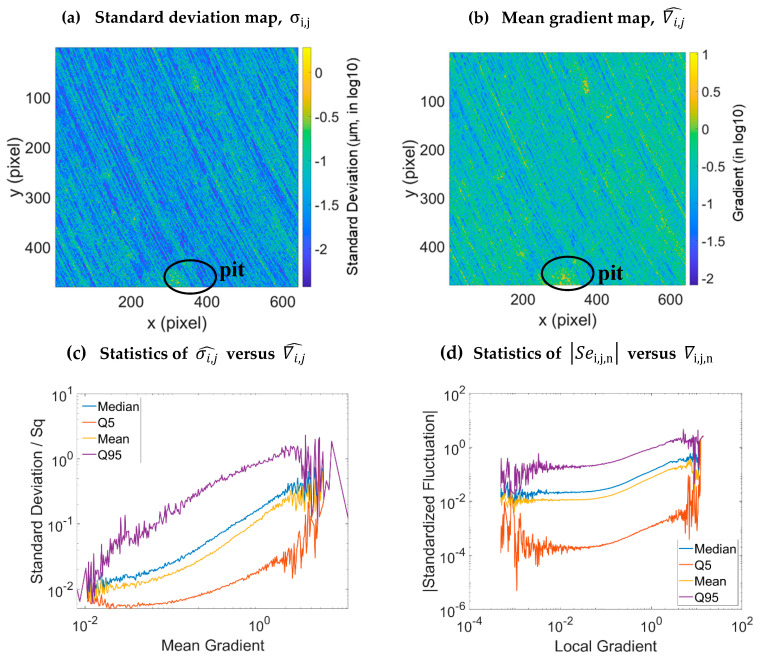
Maps and graphs calculated from the turning surface data: standard deviation map (**a**), mean gradient map (**b**), evolution of the fluctuations versus the mean gradient (**c**) and the local gradient (**d**).

Figure 3b represents the mean gradient map of the turning surfaces calculated from Equation (10). This map $\hat{\nabla_{i,j}}$ highly resembles to the map *σ_i,j_* (Figure 3a). It seems visually that these two maps are highly correlated. Equation (20) is thus plotted (Figure 3c). In log-log scale, the different curves of the global standardized fluctuations $\hat{\sigma_{i,j}}$ follow an exponential growth with the mean gradient $\hat{\nabla_{i,j}}$. This highlights the relation between the fluctuations and the surface gradient.

Fluctuation-based uncertainties are related to local surface gradients. Therefore, it is difficult to quantify measurement uncertainties without the surface topography. As a result, these top-down approaches are vital for understanding topographic measurement uncertainties.

On Figure 3d plotting individual standardized fluctuations (standardized error) versus gradients, the same tendency is observed but with a plateau for low gradients over three decades from 10^−7^ to 10^−4^. There is a critical gradient under which the fluctuations no longer depend on the gradient. However, the fluctuation range remains high for three decades (5th percentile of 10^−4^, a median of 10^−2^ and a 95th percentile of 10^−1^ then a variation of 10^−3^ (Q_95_–Q_5_)). Due to large variations of the standard deviation, logs of fluctuations are almost constant, showing that fluctuations’ variations are proportional to median fluctuations.

**Figure 4:**
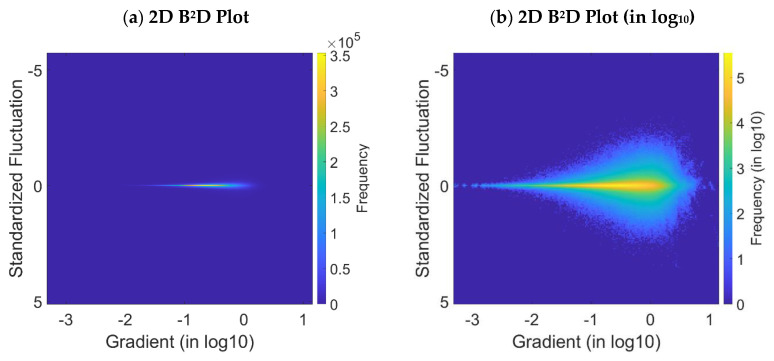
B^2^D plot of the turning surface: surface response in 2D (**a**) and 2D surface response with density expressed in log (**b**).

**Figure 5:**
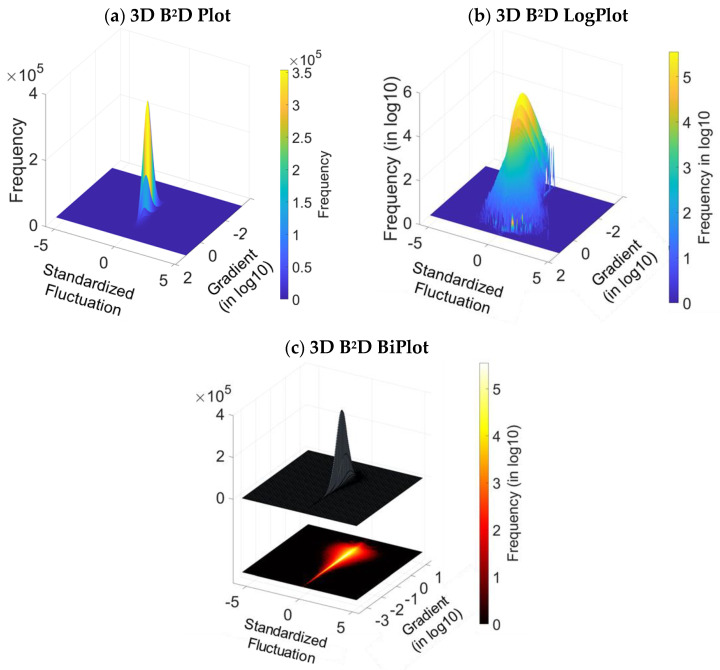
B^2^D plot of the turning surface: 3D empirical histogram (**a**), fitting with density expressed in log (**b**) and 3D surface response with density expressed in log (**c**).

The B^2^D plots are drawn from the Equation (16). Figure 4a,b show the 2D B^2^D plot with linear frequency scale and the log 2D B^2^D plot with log frequency scale against the standardized fluctuations and the log gradients, respectively. The frequency represents the number of occurrences of the standardized fluctuations versus the log gradients. The histograms in Figure 5a,b are similar to Figure 4a,b, but in 3D. Finally, Figure 5c is the histogram with its density projected in log, and it gives the final B^2^D plot representation.

The visualization of the gradient concentration is possible thanks to Figure 4a. The fluctuations are symmetrical, centered and, with respect to null values, a high density of a quasi-zero fluctuations. The symmetry shows that these amplitude fluctuations are not biased. The continuous shape of the fluctuations plot means that there is no z-offset between each map measurement, nor any recalibration after each measurement. However, visually, the fluctuations range progresses according to a continuous exponential growth with the log of the gradients.

Despite that, a question is left open about functional field of surface finishing. Manufactured surface topographies are adequately characterized by amplitudes alone. Gradients are better characterizations. Indeed, many physical models’ formulations include differential operators, such as $\frac{\partial z}{\partial x},\;\frac{\partial z}{\partial y},\;\frac{\partial^{2}z}{\;\partial x^{2}},\frac{\partial^{2}z}{\;\partial y^{2}}$. For example, friction depends on asperities’ shapes and their gradients, which form angles of attack, or on rake angles for characterizing cutting-like interactions with a counterface. Often, greater gradients provoke greater physical responses. Fluctuations are important because they correspond to gradients. Therefore, fluctuation-based uncertainties are problematic for functional characterization of, and for numerical modelling on, measured surfaces.

**Figure 6:**
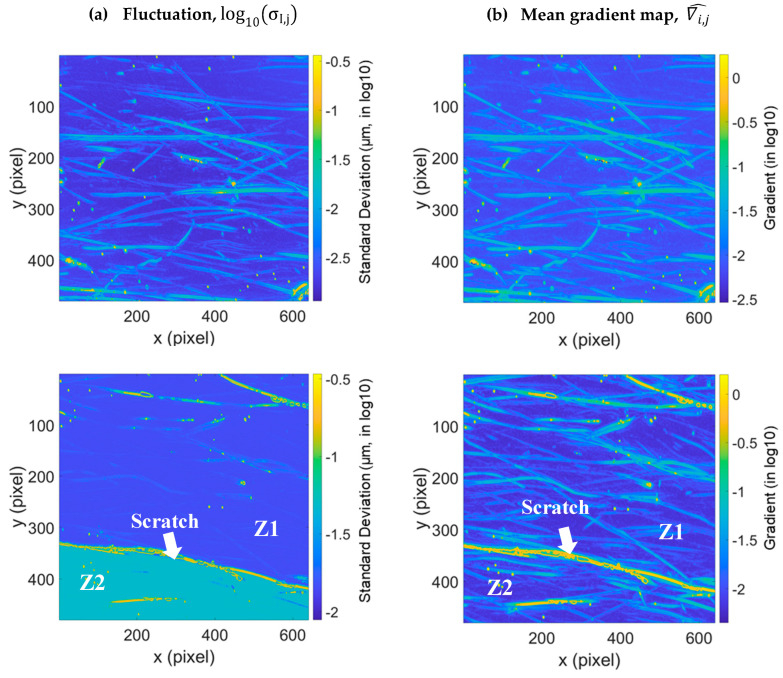
Errors projected on the 3D topographies of the two measured positions of the ophthalmic lens (**a**) and mean gradient map (**b**).

**Figure 7:**
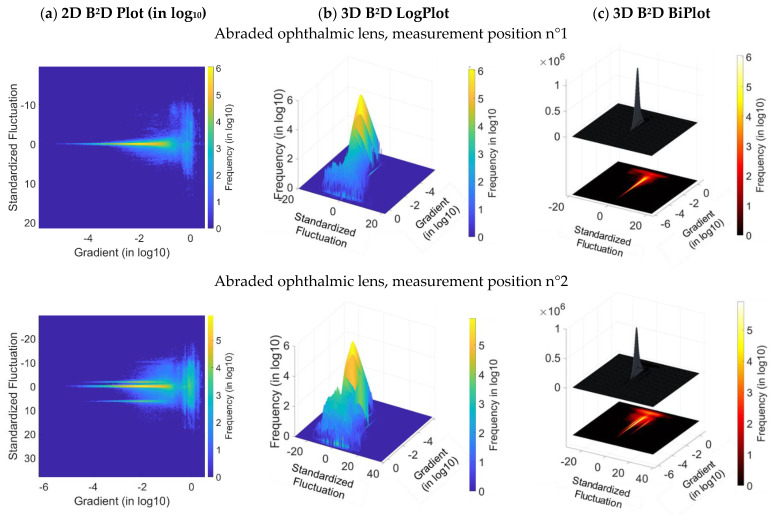
B^2^D plot for the two measured positions of the ophthalmic lens: 2D B^2^D plot in log (**a**), 3D B^2^D plot in log (**b**) and 3D B^2^D biplot (**c**).

Finally, this method is applied to the ophthalmic lens in Figure 6 and Figure 7. A projection of fluctuations and mean gradients is presented in Figure 6a,b, respectively. More computation graphs are shown in Appendix A.3 and Appendix A.4. Lemesle et al. [2] highlighted two zones which are separated by an uncertainty border (Z1 and Z2 in Figure 6). The separation of these zones is not due to their gradients, because these two zones do not appear on the gradient map (Figure 6b).

Through an analysis of B^2^D plots (Figure 7a,c), an offset is observed on the probability density with bimodal aspect in the direction of the fluctuations. More precisely, at the second position on the Figure 7a, a multiplication of 2D B^2^D plot modes can be observed due to a problem that arose during the surface measurements. This mode multiplication is caused by four topographical maps of the measurement set. Appendix B.5 shows the difference between these four maps and the other maps in the same measurement set on the roughness parameter control charts. Moreover, the histogram of fluctuations $\hat{\sigma_{i,j}}$ puts forward a bimodal structure [2]. In the direction of the high gradients on the B^2^D plot, there is a ‘new’ probability density highly extended in fluctuation but centered on the null value corresponding to gradients superior to 10^−1^ (Figure 7b). Therefore, the zone reflects high gradients due to a multitude of scratches created by the abrasive grains on the multilayer lens. The amplitude of this second histogram compared to the first one (inferior to 0.1) reflects the fraction of damaged zones with high fluctuation. The most flagrante surface feature is the boundary between the two fluctuation zones defined by the high amplitude scratch. An interpretation could be provided from the measurement system. In fact, it should be noted that the multilayer and transparent lens is measured with an interferometric microscope. The high amplitude scratch has locally modified the thickness of the layers distinctively. The hypothesis of a complex light multi-reflection in this groove and the multilayers can be a potential interpretation [25].

A comparison can be made between the lowest fluctuation plateau of the two fluctuation zones and Figure 6a of the second lens position. It shows that the fluctuation level is estimated to 10^−2.5^ = 0.0032 µm (3.2 nm), then a factor of 6 and 10, respectively, with respect to the scratch with no large amplitude. This observation shows that the complexity of the interaction between the measuring system and that the Uncertainties are Topographically and Material Dependent (UTMD). Leach et al. [26] made the attribution in their 2021 paper on metrological characteristics. It seems unreasonable to envisage a mathematical physical model that details this phenomenon which has not been encountered in other measurements performed on the biplane lens. Therefore, another top-down approach seems to be responsible for the manifestation of this optical disturbance of interferometer system on a UTMD surface with complex abrasion mechanisms. This optical artefact could introduce errors in the calculation of roughness parameters, or even a bias which would diminish the understanding of the different tribological phenomena responsible for the damage.

Liu et al. [27] proposed a model to estimate the measurement uncertainty caused by surface gradient for a white light interferometer. Within the measurement range of one single CCD (Charge-Coupled Device) pixel, height differences of the workpiece cannot be resolved laterally. Measurements are influenced by light coming from the specified range. The coherent light also results in a speckle pattern. The effect of the continuum of light illumination is calculated numerically. It is treated as *N* scattering regions, where *N* is a large number. The idea of Liu et al. [27] is to use the works of Pavliček and Soubusta [28], which created a model of uncertainties on a pixel due to scattering on a ‘flat’ rough surface to tilt the reference plane and analyze the effect on the tilt amplitude. Pavliček and Soubusta [28] therefore found a relation for the measurement uncertainties, *δ_z_* (Equation (23)).

(23) $$\delta_{z}=\frac{1}{\sqrt{2}}\sqrt{\frac{\left\langle I\right\rangle }{I}}\sigma_{h}$$

where $\left\langle I\right\rangle$ is the mean speckle pattern intensity, *I* is the individual speckle intensity, and *σ_h_* is the standard deviation of the rough surface height distribution (Equation (24)) with the assumption described in Equation (25), where *l_c_* is the coherence length.

(24) $$\sigma_{h}=\sqrt{\frac{1}{N-1}\sum_{j=1}^{N}{\left(z_{j}-z_{0}\right)}^{2}}$$

(25) $$\sigma_{h}<\frac{1}{4}l_{c}$$

Pavliček and Hýbl [29] proposed using a CCD camera and a LED with a central wavelength of 850 nm and a FWHM (Full Width at Half Maximum) spectral width of 40 nm for the interferometric measurements. For this light source configuration, the coherence length lc is therefore equal to 4.8 µm from Equation (26).

(26) $$l_{c}≅\frac{\sqrt{ln2}}{\pi }\frac{{\lambda_{0}}^{2}}{FWHM}$$

After analytical computation in a profile (i.e., in 2D), Liu et al. [27] found Equation (27) without fixing a density probability function on the amplitudes *z_i_*. The only formulated geometrical assumption is that the reference plane has no uncertainties and the height amplitude distribution of *z_i_* is symmetric about the reference plane.

(27) $${{\sigma \prime }_{h}}^{2}={\sigma_{h}}^{2}+\frac{1}{12}d^{2}{tan}^{2}\theta$$

where *d* is the pixel size and *θ* is the tilt angle. *dtan*(*θ*) is considered as the numerical gradient of the function.

They therefore proposed a 3D extension (Equation (28)).

(28) $${{\sigma \prime }_{h}}^{2}={\sigma_{h}}^{2}+\frac{1}{12}\left[{\left(\frac{\partial z_{m}}{\partial x}∆x\right)}^{2}+{\left(\frac{\partial z_{m}}{\partial y}∆y\right)}^{2}\right]$$

They found the final new expression of a pixel uncertainty (Equation (29)), i.e., pixel located in (*x*, *y*) of the topographic map, by replacing *σ_h_* by *σ’_h_* in Equation (23).

(29) $${\delta \prime }_{z}=\sqrt{\frac{1}{2}\frac{\left\langle I\right\rangle }{I}\left({\sigma_{h}}^{2}+\frac{1}{12}\left[{\left(\frac{\partial z_{m}}{\partial x}∆x\right)}^{2}+{\left(\frac{\partial z_{m}}{\partial y}∆y\right)}^{2}\right]\right)+{\delta_{r}}^{2}}$$

where *δ_r_* is the random uncertainty introduced by the environmental issue.

Local uncertainties in (*x*, *y*) are explained through Equation (29) for a proportional relation with the local surface facet gradients by neglecting environment effects. This method tends to establish a relation where uncertainties increase with gradients on a pixel. A robust statistic has to be found to confirm or disconfirm the linearity of the uncertainty/gradient relation.

## 4. Optics-Based Explanations behind the Assumed Relationship between Fluctuations and Local Gradients

### 4.1. Formulation

If the fact that the pixel is square is included, then ∆x=∆y=∆s. Assuming the gradient becomes large enough that the terms ${\sigma_{h}}^{2}$ and ${\delta_{r}}^{2}$ become negligible, Equation (30) is obtained.

(30) $${\delta \prime }_{z}=\sqrt{\frac{1}{2}\frac{\left\langle I\right\rangle }{I}\left({\sigma_{h}}^{2}+\frac{1}{12}\left[{\left(\frac{\partial z_{m}}{\partial x}∆x\right)}^{2}+{\left(\frac{\partial z_{m}}{\partial y}∆y\right)}^{2}\right]\right)+{\delta_{r}}^{2}}$$

A relationship between the local mean uncertainty $\sigma_{i,j}$*,* and the average local gradient $\hat{\nabla_{i,j}}$ of pixel (*i*, *j*) is obtained in Equation (31).

(31) $$\sigma_{i,j}=\sqrt{\frac{1}{2}\frac{\left\langle I\right\rangle }{I}\left({\sigma_{h}}^{2}+\frac{∆s^{2}}{12}{\hat{\nabla_{i,j}}}^{2}\right)}$$

Equation (29) is obtained by Liu et al. [27] and according to research by Pavliček and Soubusta [29]. Pavliček and Soubusta assume that the object’s surface is rough and the diffraction-limited point-spread function of the imaging system is broad by comparison with the microscopic surface variations, i.e., the microstructure of the surface is not laterally resolved by the imaging system. This causes the object wave to consist of contributions from many independent scattering regions. However, when the tilt method from Liu et al. is used, a correlation is introduced between scattering regions in the pixel that is not taken into account by Pavliček and Soubusta. Factor 12 in Equation (28) is also based on pure regular geometry assumptions, not statistical ones. Finally, in the case of fractal surfaces where gradient depends on the scale, the cut off between roughness of the surface and roughness of the facet in the scattering region is not really separated. For all of these reasons, we propose to include these effects by weighting the gradient effect with a factor, *c*, which leads to the final formula (Equation (32)).

(32) $$\sigma_{i,j}=\sqrt{\frac{1}{2}\frac{\left\langle I\right\rangle }{I}\left({\sigma_{h}}^{2}+\frac{c∆s^{2}}{12}{\hat{\nabla_{i,j}}}^{2}\right)}$$

### 4.2. Application

**Figure 8:**
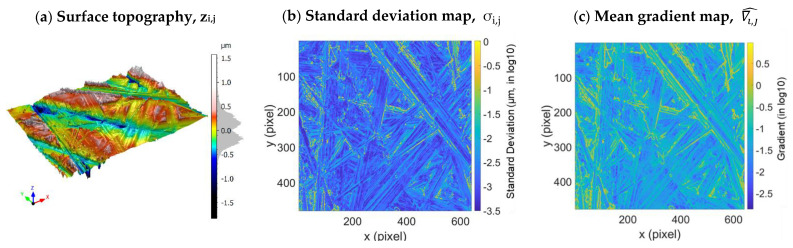
Maps calculated from the ground TA6V surface data: surface topography (**a**), standard deviation map (**b**), and mean gradient map (**c**).

The method was applied to a fractal surface (fractal dimension of 2.3) obtained by grinding a TA6V specimen with a grade 80 SiC paper. A zone of this specimen was measured 100 times by SWLI (Zygo NewView™ 7300) with a 50× lens over a range of 140 µm × 105 µm, ∆s = 0.21 µm (Figure 8). The resulting *S_q_* is 0.36 µm.

**Figure 9:**
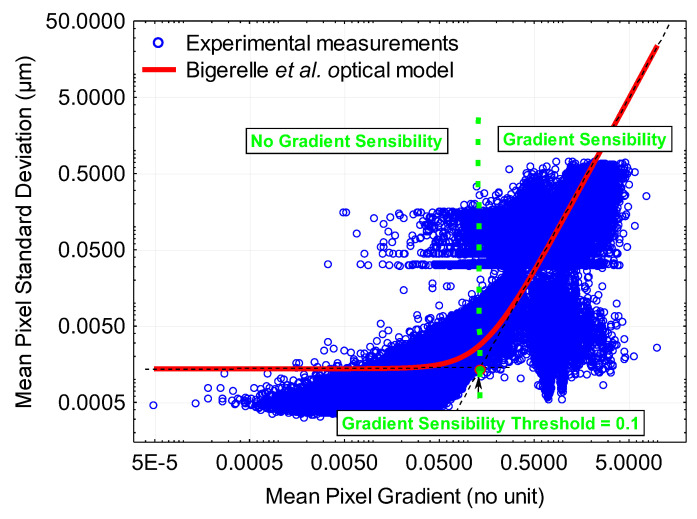
Mean pixel standard deviation versus mean pixel gradient (the red solid line corresponds to Equation (32)).

Using non-linear regression, we can calculate the values of $\frac{\langle I\rangle }{I}$, *σ_h_* and *c* (Figure 9). The model fits the experimental data quite well. For $\hat{\nabla_{i,j}}$ values below 0.1, there is no sensitivity of uncertainties to the surface gradient. Fluctuations are governed by the *σ_h_* value of 3 nm (Liu et al. [27] found a value of 2 nm). For $\hat{\nabla_{i,j}}$ values superior to 0.1, fluctuations are governed by the gradient $\frac{c{∆s}^{2}}{12}\hat{\nabla_{i,j}}$. The *c* value of 0.2 is found and shows that the effect of the gradient must be minimized by 80% compared with Liu’s initial model and, finally, $\frac{1}{2}\frac{\left\langle I\right\rangle }{I}=213$.

## 5. Discussion

The B^2^D plot is a visual tool which gives a quick visual representation of data reliability. The B^2^D plot augments traditional quality maps, because it improves the vision of surface topographies through another point of view. Therefore, this tool can guide measurement parameter selection, especially where local gradients are high. The method of gradient calculation used here is quite basic.

Other calculation methods could be performed to see how they describe correlations between uncertainty and gradient maps. This is why it will be necessary to build quality indicators to determine if a B^2^D plot, calculated with method “X”, is better than another with “Y”. This requires different surfaces for testing the calculation efficiency. The use of numerical simulation for generating surfaces can potentially be a suitable tool to test the appearance of a typical pattern in the B^2^D plot, such as the role of a z offset on each map, the effect of a spatial correlation over time, the effect of a x/y offset during multi-map acquisition, the addition of white noise, waviness, and tilt. The influence of artefacts/uncertainties calculated by numerical algorithms from the metrological domain could be used to assess on B^2^D plot. For example, simulation of the tactile measurement with a variable stylus curvature radius. This approach could then allow introduction of the bottom-up approach in the top-down philosophy.

The effect of adding non-measured points on the construction must also be studied by simulation. Indeed, the probability that the measuring system of the apparatus considers a point to be not measurable depends on each apparatus and, obviously, on the internal algorithm of height calculation, which could introduce a threshold function. The effect of missing data has not been examined in this study although it is considered in the statistical calculations; missing data could be problematic for different algorithms used for gradient calculation, for example.

Moreover, a paradox can emerge because the more a surface is ‘noisy’ or has a topography with strong local gradients, the higher the probability of obtaining NaN on the multi-map. A bias thus appears regarding the number of data points, i.e., *z_i,j_*, with high gradient decreases; instead, it should increase with an average increase of the local gradients. This bias can be significant and causes a problem when calculating roughness parameters on surfaces with NaN. In addition to any problem of roughness parameters calculation, the result will also be biased. The use of a ‘smooth’ function for filling points would only amplify this bias by adding smoothed data when the data must be rougher. This is a real challenge in the world of surface topography. Once these analyses have been performed, it will therefore be useful to build indicators which will help to interpret uncertainties and to set the apparatus using a top-down approach.

## 6. Conclusions

The presented method is built to quantify the uncertainties and make the link between them and the surface gradients. A visual graphic tool, the B^2^D plots in 2D and 3D, is developed to highlight measurement anomalies and build quantifiable indicators. In addition, according to this visualization tool, an aid for setting measurement apparatus is possible through a hundred measurement repetitions at the same position. The measurement aberrations could be distinguished with the control cards made on MountainsMap 9.3 (Appendix B). The final results can be summarized in the following five points:

1. The nature of optical measurements of irregular topographies is such that fluctuation-based uncertainties observed in repeated height measurements at one location vary with respect to the height gradients.
2. Fluctuation maps, which are based on repeated measurements, and maps of the logs of height gradients appear similar which indicates that they are correlated spatially.
3. The highest fluctuation-based uncertainties correspond to signatures of the tool/material interaction for the turned surface, and to regions with the maximum plastic deformation for the sandblasted surfaces.
4. Fluctuation-based uncertainties for an abraded surface depend on both abrasion and on light/sub-layer interactions.
5. Topographic regions which characterize surface functionality, or integrity, have the highest fluctuation-based uncertainties in their height measurements at individual locations in a measurement.

This paper opens another future possibility about the integration of uncertainties on roughness parameters. If, currently, uncertainties integration calculations could be trivial for simple roughness parameters, like *S_q_*, it could be tough to make the integration for others, like motif parameters.

A huge database is currently developed including different types of surface topography to apply and to assess with others’ methods. A software solution is in development to list and store all surface topographies related to our laboratory into the database.

## Data Availability

Not applicable.

## Nomenclature

Abbreviations	
B^2^D	Bounded Bivariate Density
NaN	Non-measured points
SWLI	Scanning White Light Interferometry
UTMD	Uncertainties are Topographically and Material Dependent
WLI	White Light Interferometry
Surface characterization parameters	
$R_{a}/S_{a}$	Arithmetic average roughness (2D/3D)
$R_{q}/R_{q}$	Root mean square roughness (2D/3D)
$S_{dq}$	Root mean square gradient (3D)
Measurement information	
A	Measuring instruments
C	Conditions of measurement
M	Set of measured maps (multi-maps)
N	Number of topographic measurements
S	Surfaces
X	Locations of measurement
Measurement parameters	
Π	Form removal polynomial
i	Coordinates of a surface point along the *x*-axis
I	X size of the measured surface topography
j	Coordinates of a surface point along the *y*-axis
J	Y size of the measured surface topography
n	Number of the measured surface topography
x	X position in a surface
y	Y position in a surface
z	Height of a surface point
$l_{c}$	Coherence length
$\lambda_{0}$	Central wavelength
Fluctuation/gradient parameters	
δ_i,j,n_	Localized uncertainty in the map *n* at the location (*i, j*)
δ_z_	Measurement uncertainties
δ_r_	Random uncertainty introduced by the environmental issue
μ	Mean height
σ	Fluctuation, i.e., standard deviation of the height
σ_h_	Standard deviation of the rough surface height distribution
$\hat{\sigma }$	Normalized fluctuation
∇	Gradient of the normal to the surface
$\hat{\nabla }$	Mean gradient
Q_q_	Moments of all gradients
$\hat{Q_{q}}$	Moments of the mean gradient
Se	Standardized fluctuations
c	Gradient effect factor
Spatial representation of gradient	
$\vec{x},\vec{y},\vec{z}$	Cartesian coordinate system
$\nu_{i,j,n}$	Vector normal to the facet
$\alpha_{i,j,n}$	Angle between *z* axis and the vector normal to the facet
Statistical parameters	
p	Probability density
Q5	5th percentile
Q50	Median
Q95	95th percentile

## Appendix B. Temporal Evolution of Roughness Parameters

In this publication, the evolution of variability in topographic maps is analyzed. It would be relevant to study roughness parameters defined in the ISO 25178 which is a standard providing guidelines for the characterization of surface texture using areal (3D) methods, taking into account the lateral (horizontal) and vertical aspects of the surface. It aims to provide a comprehensive and standardized approach for characterizing the topography of surfaces at a micro and nanoscale level. Some parameters from the standard are here studied:

- Height parameters: these parameters describe the vertical characteristics of the surface. The well-known parameter, Sa (arithmetic mean height of a surface), is studied. It represents the average deviation of the surface profile from the mean line within the evaluation length.
- Spatial parameters: these parameters evaluate the spatial arrangement and distribution of surface features. They provide information about the spacing, orientation, and density of surface irregularities. *S_al_*, autocorrelation length, is studied for characterizing surface texture. It provides information about the spatial correlation or the average distance over which surface height values are correlated. A longer autocorrelation length indicates that height values on the surface are correlated over larger distances, suggesting the presence of long-range patterns or structures. On the other hand, a shorter autocorrelation length indicates more localized or short-range roughness features. *S_tr_*, texture-aspect ratio, is also studied. It corresponds to the ratio between the characteristic wavelength of a texture and its aspect ratio. It quantifies the anisotropy or elongation of texture features on a surface. The characteristic wavelength is determined using a Fourier transform analysis of the texture profile, and the aspect ratio represents the elongation or stretching of the texture features. In simpler terms, the Texture-aspect ratio describes the elongation or stretching of texture patterns on a surface. A higher Texture-aspect ratio indicates a more elongated or anisotropic texture, while a lower ratio represents a more isotropic or uniformly distributed texture.
- Hybrid parameters: they combine both height and spatial aspects to provide a more comprehensive description of the surface texture. The *S_dr_* parameter (developed interfacial area ratio) is observed. This is expressed as the percentage of the definition area’s additional surface area contributed by the texture as compared to the planar definition area. The *S_dr_* value of a completely level surface is null.
- Feature parameters: in the context of surface texture analysis, they refer to specific measurements that describe particular aspects or characteristics of surface features. These parameters provide quantitative information about specific types of irregularities or patterns present on a surface. Watershed segmentation by Wolf Pruning is used to divide the topography into hills and compute *S_pd_* and *S_pc_* parameters. *S_pd_*, density of peaks, refers to the concentration of peaks in the surface texture of a material. It provides a measure of how closely spaced or abundant the peaks are on the surface. It can be expressed as the number of peaks per unit area or per unit length, depending on the analysis method used. *S_pc_*, arithmetic mean peak curvature, represents the arithmetic mean of the principal curvature of the peaks on the surface.
